# Book Reviews

**Published:** 1892-10

**Authors:** 


TRANSACTIONS OF THE MEDICAL SOCIETY OF THE STATE OF NEW YORK FOR THE YEAR 1892. Edited by FREDERIC C. CURTIS, M. D., Secretary. Octavo, pp. viii.—540. Published by the Society. William J. Dornan, Printer.

The transactions of this ancient and honorable society are always of an interesting nature. The one before us is no exception to the rule; in some respects it possesses advantages that none of its predecessors could claim. This might be reasonably anticipated, because each year evolves some newer and more interesting chapter in the history and progress of medicine; but one of the particular reasons for the excellent character of this volume is found in the fact that the young and enterprising president, together with his group of able assistants, succeeded in attracting to the meeting men who have contributed a vast amount of useful knowledge to the science of medicine, a valuable indication of which is found between the covers of this book.

The president, Dr. A. Walter Suiter, of Herkimer, N. Y., chose for the subject of his anniversary address, Public Health and Some of the Inconsistencies of the National Government. Dr. Suiter, in the course of his remarks, put forth an earnest plea for the establishment of a National health service. His argument is strong, his diction clear, and his conclusions are worthy of consideration. The address has been distributed in pamphlet form, and we trust it will be examined carefully by sanitarians and public health officers everywhere throughout the country.

The inaugural address of the president is a model in its make-up, and in the clear and forcible manner in which it deals with the several questions that pertain to the welfare of the profession within the State.

There are forty-eight distinct titles in this book, besides fifty-four pages of preliminary matter, and 129 pages, in which are recorded memorials and obituary notices, lists of officers, members, and delegates, members of the County Medical Societies, and an appendix of new laws relating to the medical profession in the State of New York. Every progressive physician residing within the boundaries of the Empire State should not fail to obtain the annual volumes of this society, for they are a record of the rise and progress of the profession of medicine within the borders of one of the foremost states of the union, considered, of course, at this time from a medical standpoint solely.

The book shows careful editing in every paragraph, and is a model of perfection in its mechanical creation.

---

THE MEDITERRANEAN SHORES OF AMERICA; OR, THE CLIMATIC, PHYSICAL, AND METEOROLOGICAL CONDITIONS OF SOUTHERN CALIFORNIA. By P. C. REMONDINO, M. D., Member of the American Medical Association; of the American Public Health Association; of the State Board of Health of California; Vice-President of the California State Medical Society, and of the Southern California Medical Society. Illustrated with forty-five engravings and two double-page maps. In one handsome royal octavo volume, 176 pages. Extra cloth, price, $1.25 net; cheaper edition, bound in paper, price, 75 cents net. Philadelphia: The F. A. Davis Co., publishers, 1231 Filbert street.

In his preface, Dr. Remondino says:

Southern California climatology is quite a study; many of its meteorological results are real puzzles,—puzzles met with nowhere else. * * * For instance, one of the greatest peculiarities, or oddities, of this climate consists in the relative conditions existing between the degrees of temperature and the degrees of atmospheric humidity. As a medical visitor once observed to the writer: With a rise in the thermometer you have a double discounting diminution in your humidity, and with a fall of your thermometer, you have an equal double discount of increase in the humidity, which at once produces equability. I do not know a region of the world that is so favored.

It is easy to understand that Remondino is an enthusiast in favor of Southern California as a resort for invalids, and especially those with consumptive tendencies. Particularly does he favor its littoral region. Recognizing the fact that a variety of climate is a necessity for invalids of the same disease and temperament, he shows that the region of San Diego Bay alone furnishes no less than four characteristic localities, while at Los Angeles, Dr. Remondino has seen delicate ladies and children walk the streets without even the protection of a sun-shade, "without discomfort, enervation, or resulting accident," when the thermometer stood 100 degrees in the shade.

Perhaps the profession will find the introduction the most interesting part of the work, it being largely a *résumé* of the various opinions of distinguished physicians relative to climatic conditions and localities for patients afflicted with phthisical, rheumatic, or other enfeebling diseases. While admitting the advantages of elevated regions in such cases, Dr. Remondino favors pure sea air. He thinks the censure bestowed upon its humidity undeserved, though he fully recognizes the danger from earth moisture, with its injurious germs and ferments.

The body of the book is made up largely of statistics, many in a tabulated form, and descriptions of the various ports of the country, with many beautiful illustrations and views in wood-cut and process printing, all bearing on the author's claim for the superiority of Southern California over all other healing and health-preserving places in the world. Whether, in his sanguine nature, he claims too much, must be submitted to the judgment of learned and experienced specialists. In any case, the book is an important and valuable addition to the medical literature of the day.

---

AN ILLUSTRATED ENCYCLOPEDIC MEDICAL DICTIONARY. Being a dictionary of the technical terms used by writers on medicine and the collateral sciences, in the Latin, English, French, and German languages. By FRANK P. FOSTER, M. D., editor of the *New York Medical Journal*, assisted by eleven collaborators. Vol. III. Fasc —minj. With illustrations. Quarto, pp. 775. New York: D. Appleton & Co. 1892.

The third volume of Foster's Dictionary carries the work forward to the word "Minjak-lagam," the definition of which is given as "A product of certain dipterocarpaceous plants, and analogous to gurjun balsam." The work is continued in the same style and under the same plan as originally begun. It is, altogether, the most comprehensive, elaborate, and correct medical dictionary which has been published in English, or, for that matter, in any other tongue in the world. It may be appealed to with the certainty, as near as anything can be certain, that the word desired will be found; and further, that its orthography, pronunciation, and definition will be correctly recorded. There are a number of excellent illustrations scattered through the volume, which serve to accentuate certain subjects, and which relieves the book from that monotony which would otherwise pertain to such a voluminous structure.

In our notice of the first volume we stated its importance, nay, even necessity; we have not changed that opinion, and we wish to reaffirm it here and now. It is a dictionary that every library must possess, every teacher must own, and every medical practitioner who can possibly command the price of the work must make haste to possess. The substantial way in which the publishers have dressed the volumes indicates that they will stand the wear and tear even of hard usage for a long time. The profession is indebted to Dr. Foster and his able associates for the manner in which they have continued their arduous labors during the several years of the preparation of these volumes. The stupendous labor which their production involves can only be appreciated by an examination of the books. We trust that Volume IV., which will close the series, may appear with as much promptness as possible. Everyone who has purchased this dictionary, with whom we have conversed, has expressed an impatient desire to get the whole work.

---

DISEASES OF WOMEN: A MANUAL OF NON-SURGICAL GYNECOLOGY, designed especially for the use of Students and General Practitioners. By F. H. DAVENPORT, A. B., M. D., Instructor in Gynecology, Harvard Medical School; Assistant Surgeon to the Free Hospital for Women; Physician to the Department of Gynecology, Boston Dispensary. Second edition. Revised and enlarged, with numerous illustrations. Philadelphia: Lea Brothers & Co. 1892.

The first edition of this work, published in 1889, has been exhausted, and this new one, according to the title page, is demanded. We expressed our judgment on the book in the JOURNAL at the time of the first edition, (page 185, Vol. XXIX., 1889,) and we have no reason to modify our views which were therein uttered. The plan of the work is a good one, and, in the main, contains careful instruction that is safe to follow. We are surprised, however, to find in this revision a retention of the illustrations of that abomination, Jenison's sound. Our judgment in regard to this instrument is such as to make us distrustful of an author who would give countenance to it. It is utterly useless for any gynecological procedure whatsoever, for it is incapable of repositing any uterus that cannot be so reposited without it, and yet, in this book, three illustrations, occupying the space of a full page, are devoted to it. The author's general ideas of treatment are good, and we commend the work to the student, who will find much valuable information between its covers. The specialist will, of course, be able to distinguish between that which is useful and that which is not in any work that may be put forth. It is a more difficult task to write a book that will be capable of imparting safe instruction to the student than to prepare a treatise that will answer the requirements of the specialist. We congratulate this author upon having measurably fulfilled the former indication.

---

THE POCKET PHARMACY, WITH THERAPEUTIC INDEX. A *résumé* of the clinical applications of remedies adapted to the pocket case, for the treatment of emergencies and acute diseases. By JOHN AULDE, M. D., Member of the American Medical Association ; of the Medical Society of the State of Pennsylvania ; of the Philadelphia County Medical Society, etc. New York : D. Appleton & Co. 1892.

The author of this book is one who has a large experience, and is competent to present it to the profession in an agreeable manner. He has done so in this book. We see no reason why it should be called a Pocket Pharmacy, unless it be for the reason that it cannot be carried in the pocket. It is one of the most convenient reference books on the subject that we have seen, and the therapeutic index is an admirable compilation. Every pharmacist should supply himself with the work, and so should every student of pharmaceutical science. The author makes his book a plea for small doses to be administered in accordance with physiological deductions. The pharmacy of today has made it possible for physicians to use less bulky remedies than our forefathers found it necessary to employ. There is no reason why we should not keep pace with the progress of chemistry, pharmacy, and physiology in regulating our doses.

---

A TREATISE ON PRACTICAL ANATOMY FOR STUDENTS OF ANATOMY AND SURGERY. By HENRY C. BOENNING, M. D., Lecturer on Anatomy and Surgery in the Philadelphia School of Anatomy ; Demonstrator of Anatomy in the Medico-Chirurgical College ; Demonstrator of Anatomy in the Philadelphia Dental College ; Lecturer on Diseases of the Rectum in the Medico-Chirurgical College, etc. Illustrated with 198 wood engravings. Philadelphia and London : F. A. Davis, Publisher. 1891.

In this book the author attempts to present a work that is serviceable both as a text-book on anatomy and a dissector, and, of course, it is especially written for students of anatomy and surgery. We are of the opinion that it is one of the best small works, hence, easily handled in the dissecting room. It is difficult to publish an abridgement of anatomy. The subject is necessarily one of magnitude, and can never be successfully condensed. Yet, in spite of all this, we must have a number of small works to fill a place for which this one is particularly intended. In this sense, we desire to record our approval of the work before us. The engravings are clear, and the printing has been well done, with plain-faced type and on good paper. The author states in his preface that it is not a compilation, but is the result of years of practical work and large experience in teaching. This is the only way for an anatomist to succeed when he poses in the rôle of an author. We presume this book will command ready sale at the hands of those students who really pursue their studies for the love of science.

---

A New Pronouncing Dictionary of Medicine. Being a voluminous and exhaustive hand-book of Medical and Scientific Terminology, with phonetic pronunciation, accentuation, etymology, etc. By John M. Keating, M. D., LL. D., Fellow of the College of Physicians of Philadelphia; Vice-President of the American Pediatric Society; Ex-President of the Association of Life Insurance Medical Directors; formerly Visiting Obstetrician to the Philadelphia Hospital (Blockley), and Lecturer on the Diseases of Women and Children; Consulting Physician for the Diseases of Women, St. Agnes' Hospital, Philadelphia; Gynecologist to St. Joseph's Hospital, Philadelphia; Editor Cyclopedia of the Diseases of Children, etc.; and Henry Hamilton, author of a New Translation of Virgil's Æneid into English Rhyme; co-author of Saunders' Medical Lexicon, etc.; with the collaboration of J. Chalmers DeCosta, M. D., and Frederick A. Packard, M. D. With an appendix containing important tables of bacilli, micrococci, leucomaines, ptomaines, drugs, and materials used in antiseptic surgery; poisons and their antidotes; weights and measures; thermometric scales; new officinal and unofficinal drugs, etc., etc. Philadelphia: W. B. Saunders, 913 Walnut street. Price, $5,00 net, cloth; $6,00 net, sheep.

There seems to be just now a tendency to dictionary making, and we are willing to record our approval of this reaction. For a number of years we were obliged to consult but one; now we may take our choice from among many. This latest candidate for popular favor has, despite its inaccuracies, many claims to recognition. It can be handled easily and referred to readily. The principal words are in display type, and the definitions in plain letters. The imperfections are chiefly with reference to pronunciation, though some are undoubtedly due to bad proof-reading. These faults will, no doubt, be corrected in future editions. The price of the book is reasonable, and we commend it to the profession, from whose experience only can its proper status be ascertained.

---

MANUAL OF PHYSICAL DIAGNOSIS, FOR USE OF STUDENTS AND PHYSICIANS. By JAMES TYSON, M. D., Professor of Clinical Medicine in the University of Pennsylvania, and Physician to the University Hospital; Fellow of the College of Physicians of Philadelphia; Member of the Association of American Physicians, etc. Philadelphia: P. Blakiston, Son & Co., 1012 Walnut street. 1891.

This neat little duodecimo volume of 136 pages is from the pen of an author who always has something to say when he writes, and says it well. The importance of a thorough understanding of the principles and practice of physical diagnosis cannot be overestimated, and it requires very much more elaboration than such a small volume can give to make the subject complete. But it becomes necessary to condense in many ways the literature of medicine, so as to bring it within the range of the eye of the busy man and student. Dr. Tyson has arranged this work admirably for the purpose intended, and it is printed with clear type on beautiful paper.

---

THE BOOK OF PRESCRIPTIONS, containing upwards of 3,000 prescriptions, collected from the practice of the most eminent physicians and surgeons, English and foreign, comprising also a compendious History of the Materia Medica, List of the Doses of all official or established preparations, and an index of Diseases and Remedies, by HENRY BEASLEY. Seventh edition. Philadelphia: P. Blakiston, Son & Co., 1012 Walnut street. 1892.

In this small volume are arranged, conveniently for reference, a vast number of prescriptions which have been formulated by many eminent men. It contains, besides this, a list of Latin words and phrases, and an index of diseases. Taken as a whole, it may be rated as the best book of formulæ that has recently been issued. It is well known to the profession through the previous six editions that have been published.

---

A TEXT-BOOK OF NURSING, for the use of training schools, families, and private students. Compiled by Clara S. Weeks-Shaw. Second edition, revised and enlarged with illustrations. New York: D. Appleton & Co. 1892.

This excellent volume is properly named; it is the only real text-book of nursing with which we are familiar. It should be adopted in every nursing school in the land. The demand for the second edition has not come as soon as the quality of its teaching would lead us to expect. The author is one of the most experienced women in the training of nurses, and speaks with a modest authority and a simplicity of expression that is not only delightful to read, but carries a forceful meaning that is easy to comprehend and remember. We commend the book to everybody interested in the training of nurses, as well as to every trained nurse.

